# The association between lymphocyte-monocyte ratio and postoperative acute kidney injury in patients with acute type A aortic dissection

**DOI:** 10.1186/s13019-022-01813-x

**Published:** 2022-04-01

**Authors:** Wenxiu Chen, Xiaochun Song, Liang Hong, Huan Xu, Yan Qian, Wenhao Zhang, Jiakui Sun, Xiao Shen, Ying Liu, Xiang Wang, Qiankun Shi, Han Liu, Xinwei Mu, Cui Zhang

**Affiliations:** 1 Department of Intensive Care Unit, Nanjing First Hospital, Nanjing Medical University, No. 68 Changle Road, Nanjing, 210006 Jiangsu People’s Republic of China; 2 Department of Intensive Care Unit, Wuhu Hospital Affiliated to East China Normal University, Wuhu, 241000 Anhui People’s Republic of China

**Keywords:** Lymphocyte-monocyte ratio, Acute kidney injury, Acute type A aortic dissection, Net reclassification index, Integrated discrimination improvement

## Abstract

**Background:**

The aim of this study was to investigate the relationship between baseline lymphocyte-monocyte ratio (LMR) and postoperative acute kidney injury (AKI) in patients with acute type A aortic dissection (ATAAD).

**Methods:**

ATAAD patients undergoing surgery in Nanjing First Hospital were enrolled from January 2019 to April 2021. Lymphocyte and monocyte were measured on admission. Multivariable logistic regression analyses were performed to explore the relationship between LMR and postoperative AKI. We also used receiver operating characteristic (ROC), net reclassification index (NRI) and integrated discrimination improvement (IDI) analyses to assess the predictive ability of LMR.

**Results:**

Among the 159 recruited patients, 47 (29.6%) were diagnosed with AKI. Univariate logistic regression analysis indicated that ATAAD patients with higher levels of LMR were prone to have lower risk to develop AKI (odds ratio [OR], 0.493; 95% confidence interval [CI] 0.284–0.650, *P* = 0.001). After adjustment for the potential confounders, LMR remained an independent related factor with postoperative AKI (OR 0.527; 95% CI 0.327–0.815, *P* = 0.006). The cutoff value for LMR to predict AKI was determined to be 2.67 in the ROC curve analysis (area under curve: 0.719). NRI and IDI further confirmed the predictive capability of LMR in postoperative AKI.

**Conclusion:**

Elevated baseline LMR levels were independently associated with lower risk of postoperative AKI in ATAAD patients.

## Introduction

Acute aortic dissection is one of life-threatening emergency diseases, which could lead to mortality and morbidity, especially acute type A aortic dissection (ATAAD) [1–3]. Emergency cardiac surgery has great curative effect on ATAAD [3–5]. However, a fraction of ATAAD patients may experience postoperative acute kidney injury (AKI), which may result in mortality and poor prognosis in ATAAD patients, even though they have undergone standard emergency cardiac surgery [6–8]. Therefore, it is meaningful to explore the useful biomarker that is able to predict postoperative AKI in ATAAD patients who undergo emergency cardiac surgery.

Previous experiments showed that inflammation could take part in structural damage of the aortic wall and the pathophysiological development of acute aortic dissection [9–13]. Lymphocyte-monocyte ratio (LMR) is a novel inflammatory biomarker. It has been proven that LMR is widely utilized in the field of multiple myeloma, soft tissue sarcomas, myocardial infarction and acute ischemic stroke [14–17]. One recent study has found that the lower levels of LMR might be associated with elevated probability of in-hospital mortality in ATAAD patients, independently [18]. Nevertheless, there is still a lack of research focusing on the relationship between baseline LMR and postoperative AKI in ATAAD patients treated with emergency cardiac surgery.

In this study, we aimed to investigate the association between the levels of baseline LMR and postoperative AKI in ATAAD patients undergoing emergency cardiac surgery. Furthermore, we also examined the ability of LMR to predict postoperative AKI by receiver operating characteristic (ROC) curve, net reclassification index (NRI) and integrated discrimination improvement (IDI) analyses.

## Materials and methods

### Participants

ATAAD patients undergoing emergency cardiac surgery were recruited from Nanjing First Hospital, Nanjing Medical University. All the patients underwent the surgery within 24 h after the onset of ATAAD. All the ATAAD patients were screened in the intensive care unit (ICU) after surgery and received standard treatments. This study was approved by the Ethics Committee of Nanjing First Hospital, Nanjing Medical University. Informed consent was obtained from participants or their legal representatives. Eligible participants were enrolled in the final analysis if they met the following criteria.

Inclusion criteria:

1. diagnosed as ATAAD;
2. treated with emergency cardiac surgery within 24 h;
3. age 18 years or older.

Exclusion criteria:

1. preoperative renal replacement therapy;
2. death within 48 h after surgery;
3. incomplete clinical data.

### Data collection

All participants enrolled in our study underwent standard assessments of demographic characteristics (age, gender and BMI [body mass index]), past medical history (hypertension, diabetes mellitus, atrial fibrillation, current smoking, coronary heart disease), clinical assessment and baseline laboratory data. Clinical assessment included acute physiology and chronic health evaluation II (APACHE II), European system for cardiac operative risk evaluation (EuroSCORE), systolic blood pressure, diastolic blood pressure, heart rate, operation procedures, blood transfusion, cardiopulmonary bypass (CPB) time, cross-clamp time, deep hypothermic circulatory arrest (DHCA) time, AKI. Baseline laboratory data included serum creatinine (Scr), total cholesterol (TC), triglyceride (TG), high-density lipoprotein (HDL), low-density lipoprotein (LDL), blood glucose and lactic acid (Lac).

### Measurement of lymphocyte-monocyte ratio

Blood cell counts, including lymphocyte counts and monocyte counts were sampled before emergency cardiac surgery on admission. Then, blood cell counts were analyzed by an auto-analyzer (XE-2100, Sysmex). LMR was calculated as Lymphocyte counts/monocyte counts.

### Definition of postoperative acute kidney injury

According to the newest consensus-based KDIGO criteria [19], postoperative AKI was defined as (1) small changes in serum creatinine (≥ 0.3 mg/dl or 26.5 mmol/l) when they occurred within 48 h; (2) a maximal change in serum creatinine ≥ 1.5 times the baseline value until postoperative day 7 compared with preoperative baseline values; (3) urine volume < 0.5 ml/kg/h for 6 h. In our study, we did not take urine volume into consideration owing to its inaccuracy, as done in the previous study [7].

### Operative technique

After general anesthesia, the arterial blood pressures of both the upper and lower limbs were monitored. For a long time, femoral artery cannulation was the primary choice in ATAAD surgery, the axillary/subclavian arteries (predominantly on the right) are alternative cannulation sites now. In case of hemodynamic instability or severe pericardial tamponade, the femoral artery and femoral vein were used for cannulation and initiation of CPB before sternotomy. Median sternotomy was performed. Right axillary artery and right atrial cannulation was used for CPB and selected cerebral perfusion. A vent catheter via the upper right pulmonary vein was inserted into the left atrium to decompress the left ventricle. Patients were cooled to a nasopharyngeal temperature of approximately 24 °C via CPB. For myocardial protection, an antegrade injection of cold cardioplegic blood solution was used. During the cooling process, the brachiocephalic arteries were exposed, and the proximal aortic surgery was undertaken. When the bladder temperature dropped to 27 °C, the brain continued to be perfused at a rate of approximately 5–10 ml/kg·min through the right axillary artery cannulation. If the right axillary artery was unsuitable for cannulation, the femoral artery was chosen for cannulation, and brachiocephalic arteries were perfused directly for cerebral perfusion during circulatory arrest [20]. The surgical alternatives were numerous, such as limited aortic resection, total aortic arch replacement and stent elephant trunk implantation in the descending aorta. The choice of surgical approach depended on the patient's condition. After the distal anastomosis was completed, systemic blood perfusion was restored, and the patient was gradually rewarmed. The proximal aortic anastomosis and other concomitant operations were carried out during the rewarming period, and then the coronary blood flow was restored. All patients were admitted to intensive care unit for routine postoperative monitoring.

### Statistical analysis

Statistical analyses were performed by R version 4.0.4 software (http://www.R-project.org/). All participants were categorized into three groups according to levels of LMR. Categorical variables were expressed as n (%) and continuous variables were expressed as means (standard deviation, SD) or medians (interquartile range, IQR). Differences in baseline characteristics between three groups were analyzed using independent sample t tests or Mann–Whitney U tests for continuous variables as well as the Chi-squared test or Fisher’s exact test for categorical variables, as appropriate. Multivariable analysis was adjusted for all potential confounders with a statistically significant association at *P* < 0.05 in univariate regression analysis. We used the violin plots to show the distribution of LMR between AKI group and non-AKI group. ROC curve analysis was performed to assess the overall discriminative ability of LMR to predict postoperative AKI and to establish optimal cutoff points at which the sum of the specificity and sensitivity was the highest [21]. The NRI and IDI were calculated to estimate the predictive value of adding LMR levels into logistic regression model and evaluate improvement in risk classification. NRI and IDI were calculated with the package nricens and PredictABEL [22]. Model 1 was adjusted for age and gender. All potential confounders with a statistically significant association at *P* < 0.05 in the univariate analysis for AKI were selected into Model 2. Model 2 was adjusted for age, APACHE II, diastolic blood pressure, Scr. A two-tailed value of *P* < 0.05 was considered significant.

## Results

**Fig. 1:**
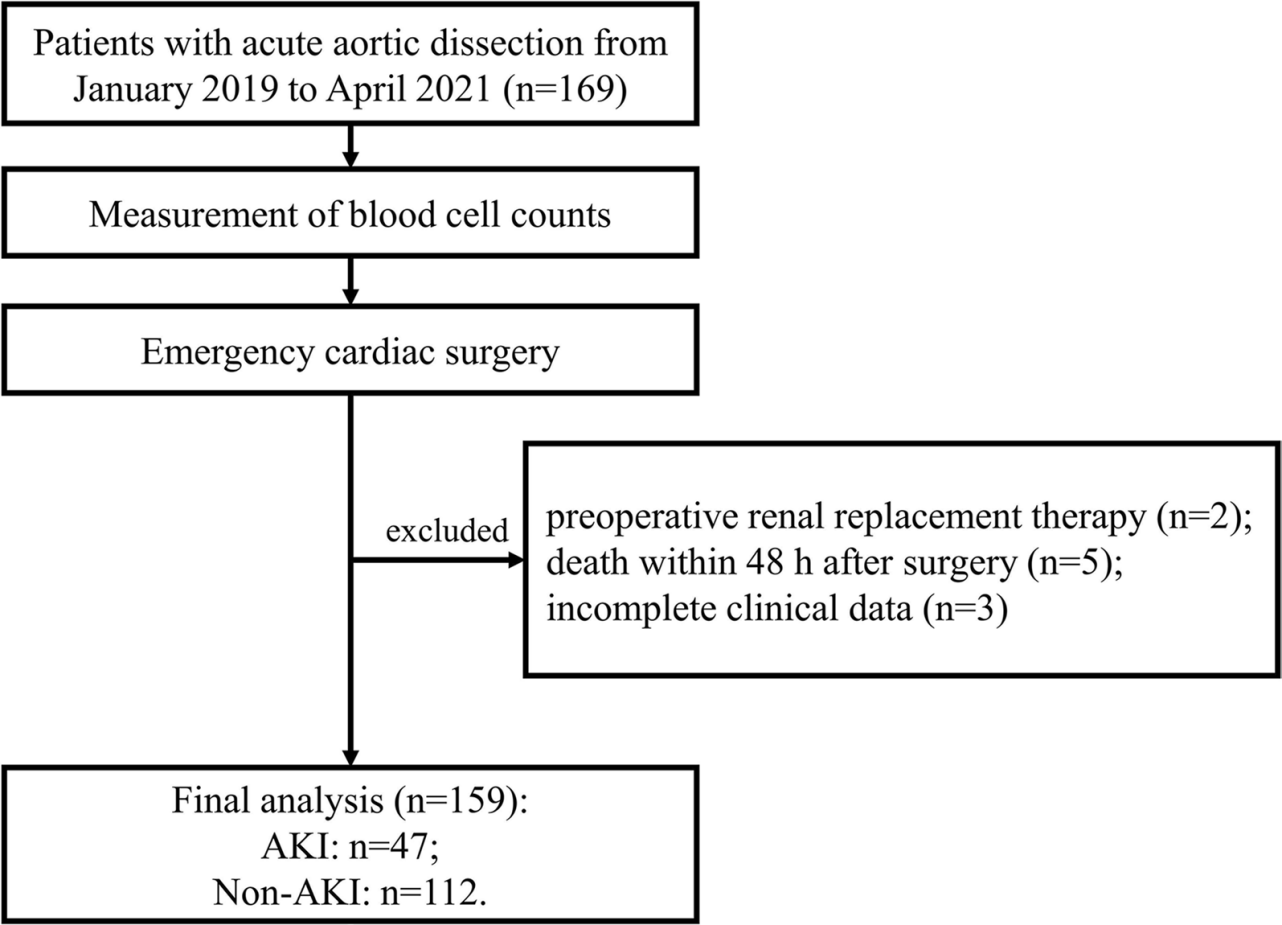
The flowchart of participants selection

**Fig. 2:**
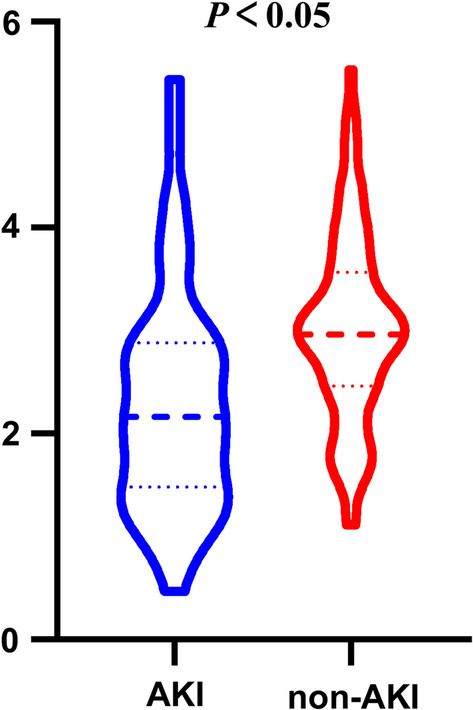
The violin plots for the lymphocyte-monocyte ratio (LMR) levels in the acute kidney injury (AKI) group and non-AKI group. * represent *P* < 0.05

From January 2019 to April 2021, a total of 169 acute aortic dissection patients treated with emergency cardiac surgery in this study (Fig. 1). Ten patients were excluded for the following reasons: preoperative renal replacement therapy (n = 2); death within 48 h after surgery (n = 5); incomplete clinical data (n = 3). A total of 159 subjects (118 men; mean age, 52.9 ± 12.1 years) were included in the final analysis. After admission, postoperative AKI was observed in 47 patients (29.6%). The median LMR level was 2.293 ± 1.082 and 3.013 ± 0.892 in patients with or without AKI, respectively. The difference in LMR between AKI group and non-AKI group was significant (Fig. 2).

Baseline characteristics of the study participants according to LMR levels were provided in Table 1. Significant differences between the three groups were described as follows: blood transfusion (*P* = 0.021), CPB time (*P* = 0.023), cross-clamp time (*P* = 0.004), DHCA time (*P* = 0.001), AKI (*P* = 0.001), Scr (*P* = 0.009), TC (*P* = 0.021) as well as LDL (*P* = 0.025).

**Table 1:** Baseline characteristics of the acute type A aortic dissection patients undergoing surgery

Variable	First tertile (n = 53)	Second tertile (n = 53)	Third tertile (n = 53)	*P*
*Demographic characteristics*				
Age, years	55.0 ± 12.0	53.5 ± 12.0	50.4 ± 11.6	0.136
Male, n (%)	41 (77.4)	35 (66.0)	42 (79.2)	0.243
BMI, kg/m^2^	25.2 (23.5, 27.4)	25.3 (22.4, 28.1)	24.9 (22.3, 28.7)	0.951
*Past medical history, n (%)*				
Hypertension	50 (94.3)	49(92.5)	48(90.6)	0.763
Diabetes mellitus	1 (1.9)	2 (3.8)	2 (3.8)	0.813
Atrial fibrillation	1 (1.9)	1 (1.9)	1 (1.9)	0.999
Current smoking	25 (47.2)	25 (47.2)	28 (52.8)	0.797
Coronary heart disease	4 (7.5)	3 (5.7)	1 (1.9)	0.398
*Clinical assessment*				
APACHE II, score	12 (10, 15)	12 (10, 14)	12 (10, 15)	0.566
EuroSCORE, score	5 (5, 6)	5 (5, 6)	5 (4, 6)	0.610
Systolic blood pressure, mmHg	142 (128, 164)	136 (125, 158)	140 (126, 158)	0.569
Diastolic blood pressure, mmHg	81 (70, 97)	71 (64, 88)	74 (67, 89)	0.060
HR, /min	78 (66, 87)	78 (70, 87)	80 (70, 87)	0.830
Total arch replacement, n (%)	38 (71.7)	43 (81.1)	46 (86.8)	0.147
Semi-arch replacement, n (%)	12 (22.6)	6 (11.3)	5 (9.4)	0.112
Stented elephant trunk, n (%)	42 (79.2)	42 (79.2)	47 (88.7)	0.338
Bentall procedure, n (%)	6 (11.3)	2 (3.8)	1 (1.9)	0.084
Ascending aorta replacement, n (%)	49 (92.5)	51 (96.2)	52 (98.1)	0.351
Blood transfusion, ml	3475 (2890, 4350)	2980 (2360, 3560)	3560 (2725, 4250)	0.021
CPB time, min	172 (153, 179)	160 (135, 170)	174 (149, 186)	0.023
Cross-clamp time, min	96 (80, 107)	85 (69, 96)	95 (80, 112)	0.004
DHCA time, min	20 (18, 21)	19 (17, 20)	20 (19, 22)	0.001
AKI, n (%)	27 (50.9)	13 (24.5)	7 (13.2)	0.001
*Baseline laboratory data*				
Scr, umol/L	87.4 (69.4, 109.2)	71.1 (54.9, 94.2)	71.7 (60.7, 95.2)	0.009
TC, mmol/L	2.64 (2.24, 3.00)	2.85 (2.41, 3.43)	2.40 (1.92, 3.15)	0.021
TG, mmol/L	1.26 (0.93, 1.55)	1.16 (0.89, 1.76)	1.37 (0.95, 2.10)	0.507
HDL, mmol/L	0.74 ± 0.19	0.73 ± 0.23	0.76 ± 0.19	0.090
LDL, mmol/L	1.27 (0.97, 1.55)	1.44 (1.07, 1.78)	1.07 (0.87, 1.56)	0.025
Blood glucose, mmol/L	7.06 (6.27, 8.04)	6.85 (6.13, 8.25)	7.54 (6.43, 8.61)	0.274
Lac, mmol/L	1.40 (0.90, 1.90)	1.00 (0.80, 1.80)	1.20 (0.80, 2.50)	0.589

BMI, body mass index; APACHE II, acute physiology and chronic health evaluation II; EuroSCORE, european system for cardiac operative risk evaluation; HR, heart rate; CPB, cardiopulmonary bypass; DHCA, deep hypothermic circulatory arrest; AKI, acute kidney injury; Scr, serum creatinine; TC, total cholesterol; TG, triglyceride; HDL, high density lipoprotein; LDL, low-density lipoprotein; Lac, Lactic acid

Table 2 illustrated the results of the logistic regression for postoperative AKI. Univariate logistic regression analysis demonstrated that age (odds ratio [OR], 1.039; 95% confidence interval [CI] 1.009–1.072, *P* = 0.013), APACHE II (OR 1.099; 95% CI 1.007–1.204, *P* = 0.036), diastolic blood pressure (OR 1.022; 95% CI 1.005–1.041, *P* = 0.017), Scr (OR 1.032; 95% CI 1.020–1.047, *P* = 0.001), and LMR (OR 0.493; 95% CI 0.284–0.650, *P* = 0.001) might be linked to postoperative AKI. After adjustment for the potential confounders, LMR remained an independently related factor with postoperative AKI (OR 0.527; 95% CI 0.327–0.815, *P* = 0.006).

**Table 2:** Logistic regression analysis for the risk factors associated with postoperative acute kidney injury in patients with type A aortic dissection

Variable	Unadjusted OR (95%CI)	*P*	Adjusted OR (95%CI)	*P*
*Demographic characteristics*				
Age	1.039 (1.009–1.072)	0.013	1.048 (1.009–1.091)	0.019
Male	0.645 (0.305–1.390)	0.254		
BMI	1.055 (0.966–1.154)	0.230		
*Past medical history*				
Hypertension	2.206 (0.553–14.729)	0.320		
Diabetes mellitus	1.615 (0.208–10.059)	0.606		
Atrial fibrillation	1.196 (0.055–12.779)	0.885		
Current smoking	0.610 (0.302–1.209)	0.160		
Coronary heart disease	0.785 (0.112–3.558)	0.772		
*Clinical assessment*				
APACHE II	1.099 (1.007–1.204)	0.036	1.063 (0.956–1.179)	0.248
EuroSCORE	1.176 (0.999–1.397)	0.055		
Systolic blood pressure	1.013 (0.999–1.026)	0.054		
Diastolic blood pressure	1.022 (1.005–1.041)	0.017	1.024 (0.999–1.050)	0.051
HR	0.999 (0.975–1.023)	0.921		
Total arch replacement	0.904 (0.398–2.166)	0.815		
Semi-arch replacement	1.050 (0.379–2.664)	0.921		
Stented elephant trunk	0.863 (0.366–2.157)	0.742		
Bentall procedure	0.667 (0.097–2.886)	0.622		
Ascending aorta replacement	1.051 (0.218–7.530)	0.953		
Blood transfusion	1.000 (0.999–1.001)	0.104		
CPB time	0.996 (0.985–1.006)	0.447		
Cross-clamp time	0.993 (0.977–1.008)	0.356		
DHCA time	0.959 (0.813–1.125)	0.610		
*Laboratory data*				
Scr	1.032 (1.020–1.047)	0.001	1.033 (1.019–1.050)	0.001
TC	0.665 (0.404–1.047)	0.092		
TG	1.371 (0.912–2.059)	0.125		
HDL	0.194 (0.031–1.067)	0.067		
LDL	0.620 (0.312–1.140)	0.146		
Blood glucose	1.009 (0.931–1.082)	0.773		
Lac	1.121 (0.911–1.392)	0.269		
LMR	0.493 (0.284–0.650)	0.001	0.527 (0.327–0.815)	0.006

BMI, body mass index; APACHE II, acute physiology and chronic health evaluation II; EuroSCORE, european system for cardiac operative risk evaluation; HR, heart rate; CPB, cardiopulmonary bypass; DHCA, deep hypothermic circulatory arrest; Scr, serum creatinine; TC, total cholesterol; TG, triglyceride; HDL, high density lipoprotein; LDL, low-density lipoprotein; Lac, Lactic acid; LMR, lymphocyte-monocyte ratio

Table 3 showed that adding LMR to model 1 ameliorated the prediction of postoperative AKI. Moreover, adding LMR to model 2, which included all the potential confounders with a statistically significant association in the univariate analysis can improve predictive power for AKI in ATAAD patients undergoing cardiac surgery.

**Table 3:** Reclassification statistics (95% CI) for postoperative acute kidney injury by LMR in patients with type A aortic dissection

Variable	Estimate (95% CI)	*P*
*Model 1 + LMR*		
NRI	0.260 (0.065–0.456)	0.009
IDI	0.110 (0.058–0.162)	0.001
*Model 2 + LMR*		
NRI	0.200 (0.041–0.360)	0.014
IDI	0.055 (0.016–0.093)	0.005

Model 1, adjusted for age and gender

Model 2, adjusted for age, APACHE II, DBP and Scr

LMR, lymphocyte-monocyte ratio; NRI, net reclassification index; IDI, integrated discrimination improvement; APACHE II, acute physiology and chronic health evaluation II; DBP, diastolic blood pressure; Scr, serum creatinine

**Fig. 3:**
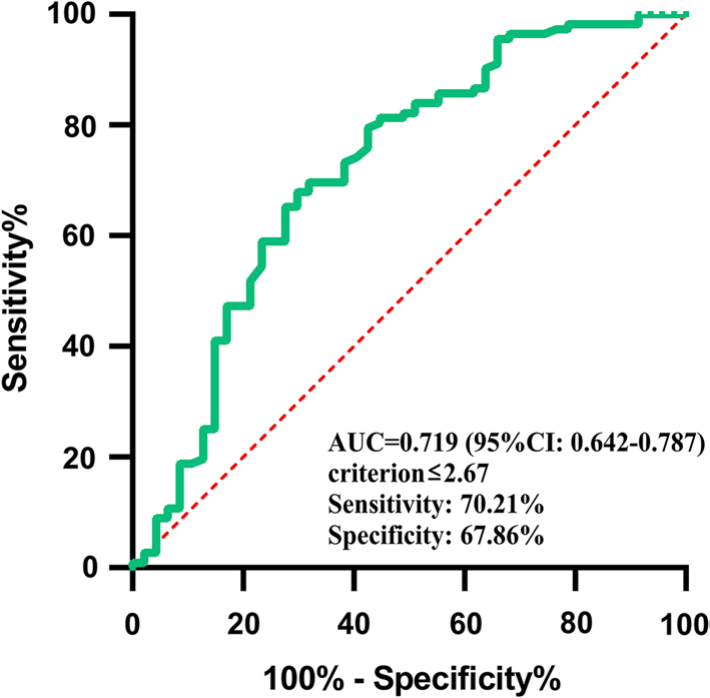
Receiver operating characteristic (ROC) curve for the value of lymphocyte-monocyte ratio (LMR) to predict postoperative acute kidney injury (AKI)

We performed ROC analysis in Fig. 3. The area under curve (AUC) of LMR was 0.719 (95% CI 0.642–0.787) with the ability to discriminate postoperative AKI. The optimal cutoff value for LMR as a predictor of postoperative AKI was determined to be 2.67 in the ROC curve analysis, yielding the largest Youden’s index value (a sensitivity of 70.21% and a specificity of 67.86%).

## Discussion

To our knowledge, this is the first study to investigate the association between baseline levels of LMR and postoperative AKI in ATAAD patients who undergo emergency cardiac surgery. The results of our study manifested that higher LMR levels were independently associated with lower risk of postoperative AKI in ATAAD patients. We also found that LMR might be able to predict postoperative AKI. What is more, adding LMR to the models consisting of the risk factors, which are independently related to postoperative AKI, could improve predictive ability for postoperative AKI.

AKI is a common complication which occur after the emergency cardiac surgery in ATAAD patients. According to the previous researches, the incidence of postoperative AKI ranged from 20 to 67% [23–28]. The incidence postoperative AKI in our study is 29.6%, which is consistent with previous researches and relatively low, perhaps because of the skilled surgical procedures and the strict postoperative management in our center.

During the development of aortic dissection, Inflammation plays an important and main role [12]. It was reported that inflammatory pathways may be related to the pathogenesis of medial degeneration, the main histological finding of acute aortic dissection. Infiltration of the aortic wall with inflammatory cells, such as lymphocytes and monocytes, could be involved in the expression of proteases and cytokines. In addition, they may also be associated with the release of reactive oxygen species as well as apoptosis of smooth muscle cells in the aortic artery, which may be able to have an impact on medial degeneration [10, 29]. LMR, which combines lymphocyte counts and monocyte counts, is calculated from blood cell counts, so it is convenient and inexpensive for clinicians to acquire the data of LMR. According to a previous study published [18], Lin and colleagues discovered that in ATAAD patients, in-hospital death may be linked to LMR, according to the results of multivariate analysis and Kaplan–Meier survival curves. In our study, we collected the data of blood cell counts before emergency cardiac surgery. Therefore, the levels of LMR are supposed to be obtained by clinicians before postoperative AKI occurs, to prove the predictive value of LMR in clinical practice.

In our single center study, we also found that age, the baseline score of APACHE II, diastolic blood pressure and baseline Scr may be independently related to postoperative AKI. Several previous researches and meta-analysis indicated that the older ATAAD patients may be easier to develop AKI after surgery [26, 30, 31]. The study directed by Hua Liu and colleagues displayed that the score of APACHE II might be connected with postoperative AKI, independently [32]. The connection between preoperative Scr and postoperative AKI was also be found in previous study [23].

There are several limitations in our study. First, the sample size of our study is relatively small, so the level of evidence provided by this study is not high enough. Second, the patients enrolled in our study are all Chinese. Accordingly, the conclusion we draw may not be able to be promoted abroad and there needs more clinical researches on the relationship between baseline LMR and postoperative AKI in ATAAD patients. Third, we did not take urine volume into consideration because of its inaccuracy, as done in the previous study, which may result in bias. Moreover, we only investigated the role of LMR in ATAAD patients, and we plan to study the role of LMR in the broader subset of open heart surgery on CPB in the future.

## Conclusions

In summary, our study indicated the association between baseline levels of LMR and postoperative AKI in ATAAD patients who underwent emergency cardiac surgery. LMR may be able to serve as a protective factor of AKI after emergency cardiac surgery. Moreover, this study might show the ability of baseline LMR in predicting postoperative AKI, due to the results of ROC, NRI and IDI analyses. What is more, further researches are required to verify these results about postoperative AKI in ATAAD patients.

## Data Availability

The datasets analysed during the current study available from the corresponding author on reasonable request.

## Abbreviations

ATAAD: Acute type A aortic dissection
AKI: Acute kidney injury
LMR: Lymphocyte-monocyte ratio
ROC: Receiver operating characteristic
NRI: Net reclassification index
IDI: Integrated discrimination improvement
ICU: Intensive care unit
BMI: Body mass index
APACHE II: Acute Physiology and Chronic Health Evaluation II
EuroSCORE: European System for Cardiac Operative Risk Evaluation
CPB: Cardiopulmonary bypass
DHCA: Deep hypothermic circulatory arrest
Scr: Serum creatinine
TC: Total cholesterol
TG: Triglyceride
HDL: High-density lipoprotein
LDL: Low-density lipoprotein
Lac: Lactic acid
